# Breaking ground: insights from a WHO-approved surgical mission amid Sudan's healthcare collapse

**DOI:** 10.3389/fpubh.2025.1707616

**Published:** 2026-01-07

**Authors:** Bilal Irfan, Shayan Ali, Roya Ardah, Zehra Jaffery, Asma Ahmed, Nada Othman, Abdelnour Ali, Syed Irfan Qasim Ali, Mohammad Burney

**Affiliations:** 1 Center for Bioethics, Harvard Medical School, Boston, MA, United States; 2 Center for Surgery and Public Health, Brigham and Women's Hospital, Boston, MA, United States; 3 Department of Neurology, University of Michigan Medical School, Ann Arbor, MI, United States; 4 Department of Epidemiology, University of Michigan School of Public Health, Ann Arbor, MI, United States; 5 Department of Neuroscience, Austin College, Sherman, TX, United States; 6 Gift of Disability Alleviation, Dallas, TX, United States; 7 North Texas Medical Research Institute, Rockwall, TX, United States; 8 Southern Methodist University, Dallas, TX, United States; 9 Sudanese American Physicians Association, Port Sudan, Red Sea State, Sudan; 10 National Center for Gastroenterology and Liver Diseases, Khartoum, Khartoum State, Sudan; 11 Department of Public Health, University of Bahri, Khartoum, Khartoum State, Sudan; 12 Kassala Teaching Hospital, Kassala, Kassala State, Sudan; 13 Wad Medani Teaching Hospital, Wad Medani, Al Gezira, Sudan; 14 Pain and Spine Physicians, Southlake, TX, United States; 15 Orthopaedic Specialists of Dallas, Rockwall, TX, United States

**Keywords:** conflict zone, emergency response, medical mission, Sudan, surgical capacity

## Introduction

Since April 2023, Sudan has been embroiled in a civil war that has resulted in one of the worst humanitarian crises of the twenty-first century (1, 2). Even prior to this war, Sudan suffered from a severe shortage of healthcare workers (HCWs) due to recurring hostilities, forced displacement, voluntary exit of skilled professionals outside of the country, and destruction of educational infrastructure—with the number of physicians, nurses, and other health workers per 10,000 people far below the World Health Organization (WHO) 22.8 healthcare worker minimum (3–6). The war has resulted in widespread damage to healthcare infrastructure (HI) and acute shortages of water and electricity, both essential for healthcare services (7). By April 2025, over 30.4 million people were in need of humanitarian assistance, and 70–80% of healthcare facilities had been closed—resulting in increased morbidity and mortality rates across the country (7). In this context, international emergency medical teams medical missions have entered Sudan to provide care to the affected population. We present insights from among one of the first WHO-approved orthopedic missions since the recent escalations in hostilities, and argue for the necessity of international medical teams of surgical specialties to respond to this conflict zone.

## Operational context and rationale for a surgical EMT in Sudan

On April 6, 2025, Gift of Disability Alleviation (GODA), a USA-based nonprofit, in collaboration with the Sudanese American Physicians Association (SAPA) and with the coordination of the WHO, launched a 10-day Emergency Medical Team (EMT) Type 2 surgical mission at the SAPA-supported hospital in Wad Madani. This deployment, to the best of our knowledge, marked one of the first WHO-approved orthopedic surgical missions to Sudan since the onset of the civil war. The mission aimed to deliver orthopedic care, conduct a site evaluation for future deployments, and support efforts to rebuild the country's surgical capacity. Complementing that operational model is the EMT-linked 4“S” surge capacity framework that organizes planning and evaluation around Staff, Systems, Supplies, and Structure or Space (8).

The rationale behind a surgical-focused EMT rotation in Sudan is not simply the extraordinary rise in trauma injuries arising from protracted urban warfare, and the burden it places on the healthcare system, but also the pre-existing deficits in Sudan's healthcare system capacity (9–12). Orthopedic injuries account for a large portion of trauma-related presentations in armed conflicts, as well as LMICs, and often lead to disability in environments where prehospital care is minimal and definitive fixation is delayed (13–17). In conflict-affected regions, the problem is often compounded by the near-absence of pathways for treatment referral, the out-migration or killing of experienced healthcare staff, and shortages of essential implants, surgical equipment, and rehabilitation resources (18–20).

The predeployment planning logic for EMTs in such environments must therefore begin with a realistic assessment of what care or provisions can be delivered safely given constraints on monitoring, oxygen, sterilization, and postoperative care. For example, the WHO and the World Federation of Societies of Anaesthesiologists have set international standards for safe anesthesia practice that emphasize monitoring, trained providers, and context-appropriate equipment (21). In environments of resource-scarcity, adherence to the spirit of these standards may require adaptation. The safe delivery of anesthesia, often essential to surgical procedures, is often made possible in austere environments from teams adapting to simple, robust, and context-appropriate strategies and relying on continuous clinical monitoring and local insights and expertise to strategize feasible care plans (22–25).

The mission was predicated on successful coordination prior to deployment and considering the clinical needs and ability to operate. SAPA established links with the WHO and as well as national and regional health ministries and actors, including humanitarian groups, in a bid to pull together and draw on different resources. An office was opened at the SAPA Al-Gazeira Hospital to manage patient pre-registration, community outreach, and logistical planning to ensure that the facility was ready for operation. This groundwork ensured triage stations, patient intake systems, and essential utilities were prepared to allow for EMTs to start services immediately upon arrival (26).

## April 2025 mission and the need for successive EMT rotations

SAPA operationalized a hospital in Wad Madani in advance of GODA's arrival in April 2025, laying foundational infrastructure that enabled rapid mobilization. SAPA physicians assisted in triage, language interpretation, and patient intake. SAPA-led triage and intake protocols enabled the application of the “Systems” component of the aforementioned 4“S” model, allowing for high-throughput evaluation and effective surgical prioritization under the constraints of armed conflict. The goals of this WHO-coordinated mission were twofold: providing surgical care to the Sudanese population and assessing healthcare infrastructure (HI) to guide future efforts. Patient information was documented manually on standardized intake forms, which included patient demographics and brief medical history. Individuals presented their X-rays as printed film or as digital images on mobile devices, a phenomenon reported in other conflict zones (27). Some laboratory services were also available for routine blood work.

The operating team was composed of one international anesthesiologist, one international orthopedic surgeon, two Sudanese anesthesia technicians, and four Sudanese surgery residents. In total, they performed 690 consultations in outpatient clinics, 29 surgeries, six interventional pain management procedures, and four castings during the mission's span (Table 1). The most prevalent injuries were proximal femur fractures (PFFs), with clinicians witnessing low rates of post-surgical infection, a common-cause of concern in war-related environments (28).

**Table 1:** Daily number of patients seen in clinic and number of surgeries performed, April 9–12, 2025.

**Date**	**Patients seen (*n*)**	**Surgeries performed (*n*)**
April 9	150	7
April 10	215	4
April 11	125	12
April 12	200	6
Total	690	29

## Staff and systems: matching skills to case mix and building capacity under duress

The “Staff” domain in the 4S framework encompasses far more than just a headcount of healthcare workers present. Sudan's conflict has accelerated the loss of experienced clinicians through frequent displacement events, internally and those leaving the country entirely, leaving gaps in perioperative nursing, anesthesia, and orthopedic care (29). Sudanese universities have documented disruptions to the administration of curriculum and teaching, loss of laboratories and learning spaces, and major impediments to clinical training for medical students from 2023 onwards (30). EMTs cannot entirely repair that structural deficit during a short deployment, yet they can target critical inflection points in skills transfer. Evidence from task-sharing and competency-based training in low-resource settings suggests that focused mentorship may be helpful and can be delivered effectively to non-specialists when supervision and clear protocols are present (31). In the operating theater, deliberate adoption of the WHO Surgical Safety Checklist may aid in strengthening team communication, reducing certain complications, and length of stay (32, 33). Furthermore, embedding infection prevention and control principles within global surgery initiatives can be utilized to realize durable gains in safety, especially when adopted as a scaffold for infection prevention and control behaviors by routine on-ground medical staff (34).

“Systems” in the 4“S” model can refer to the protocols, workflows, and governance arrangements that convert the scarcity and risk-affected present into clinically sound operations and outputs. In Sudan, several organizations have operated hospitals that often host internally displaced persons, with standardized intake forms and context-appropriate triage. Such processes matter because case selection is a powerful lever available to an EMT when monitoring and postoperative beds are constrained.

Of course, even well-structured training encounters can falter under the conditions of armed conflict. Repeated mass casualty events and curfews can disrupt schedules for healthcare staff. For example, a UNICEF report documented widespread cholera transmission in 17 of Sudan's 18 states (35). Such a reality serves to underscore the need for flexible deployment and training initiated by EMTs. It is possible that an EMT orthopedic surgeon may need to teach sterile draping and basic fracture temporization in the morning, then supervise a nurse-led checklist run-through before a block room session in the afternoon.

Medical supply chain processes into and within Sudan have often been erratic, with damaged roads, checkpoints, and intermittent flight access to areas of relative security. The consequences that emerge from this for surgery are quite grim, as without necessary surgical supplies, anesthesia, and implants, surgeons must often rely on temporary measures and *ad-hoc* treatment options. There is also a risk of infection spreading as a consequence of this (14, 19, 28, 36, 37). The implication for international EMTs is often that kit designs and procurement should prioritize ensuring sterility and the ability to monitor patients rather than necessarily a variety in instruments (though often needed). One can do fewer cases safely with sterile fields than more cases poorly with contaminated ones. WHO and allied patient-safety bodies emphasize that the surgical checklist, routine antibiotic stewardship, and availability of pulse oximetry are foundational elements of conducting a safe surgery such that even partial implementation can confer benefits when adhered to consistently (38, 39).

The “structure” domain concerns the physical environment in which care is delivered. In Sudan, surgical theaters can often lack positive pressure ventilation, restrictions on entry, or sealed doors. Movement restriction, temperature regulation where power permits, and clear demarcation of clean and contaminated zones can serve to reduce cross-contamination and reinforce good habits among rotating staff (40). In many facilities, power is intermittent and water quality is unreliable. A practical response could envisage combining choice of procedure and the necessary environmental workarounds. For example, teams can co-design pragmatic rules with local staff that limit door openings, define footwear and gowning expectations tailored to available supplies, and embed a short pause in which sterility indicators are reviewed before incision. Such micro-systems can be sustained by local teams after departure because they require not as much equipment and rely somewhat on the maintenance of norms.

## Coordination under insecurity: making the 4S framework operational

Turning the 4S framework into a daily practice may often require collaboration and communication across organizations. In Sudan, emerging partnerships between diaspora professional associations, local health authorities, and international actors could be used to ensure shared protocols and processes for triage are utilized to accelerate safe surgical environments even in areas of extreme resource-limitations. The 4S model can also incorporate a security lens that sits across domains, perhaps by adding dimensions such as security and sponsorship to classic 4S planning. These additions could speak directly to humanitarian response realities in which access, staff safety, and violence against healthcare determine whether any element of the 4S is achievable (41). In practice, this could mean that EMTs treat security assessments, convoy route, and negotiation for protected movement as part of clinical systems work rather than as separate logistics. In the same vein, sponsorship arrangements that formalize roles with local professional bodies and ministries can stabilize staffing and improve continuity for follow-up, which is otherwise a chronic Achilles' heel for short-term medical missions.

Information systems remain among one of the thinnest strands in Sudan's surgical safety net. Electronic medical records are often out of the question. Yet a paper registry that travels with the patient or is duplicated in a ward folder and an operating theater log might create just enough data to guide antibiotic stewardship, surveillance for primary complications, and referral decisions (18, 32, 42, 43).

## Discussion

The crisis in Sudan forces international EMTs to confront a sobering reality wherein a health system is facing patients presenting with trauma-related injuries, the effects of famine, cholera, and the collapse of civilian infrastructure, all of which can involve increasing the envelope of risk in surgeries, and their associated complications, in ways that no amount of technical excellence can fully neutralize. The task at hand is often not to reproduce surgical designs in high-income settings, but rather to define and deliver a somewhat constrained package of care that is safe, ethical, and system-building. Recent scholarship on health-system surge capacity and EMT capacity building offers a useful conceptual map (8). The 4S framework directs attention to the interdependence of people, processes, supplies, and space.

There are structural risks that even the best-prepared EMT cannot eliminate. Facility functionality may deteriorate due to renewed attacks on healthcare; supply corridors may close without notice; epidemics may lead to abrupt diversion of staff and resources. These risks pave the way for an argument for the regionalization of efforts and for layered sponsorship that ties EMTs to diaspora organizations and local ministries with standing presence. They can also provide a basis for an argument for humility about claims of “firsts” or singular innovations in complex conflicts where documentation is fragmented and access is uneven. The more defensible claim, and the more constructive goal, is to show that safe surgery is feasible under extreme constraints when teams adhere to crisis standards, restrict procedures to what can be done safely, and consider investing as much in systems and staff as in scalpels.

Sudan's people will need more than well-run operating rooms. They need a health system that can protect them from the next outbreak, the next famine, and the next round of violence. The EMT community cannot wholly deliver that system, but it can choose to operate as one of its builders. If EMTs consistently leave behind trained staff who use checklists, reliable micro-systems for infection control, and honest records to guide the next team, then each deployment becomes a cumulative contribution rather than an isolated act. The evidence base already points the way. The challenge is to practice within it, with discipline and respect for the limits that this conflict imposes.
